# Putative endothelial progenitor cells do not promote vascular repair but attenuate pericyte–myofibroblast transition in UUO-induced renal fibrosis

**DOI:** 10.1186/s13287-019-1201-5

**Published:** 2019-03-21

**Authors:** Juan Yang, Meng Wang, Fengming Zhu, Jie Sun, Huzi Xu, Octavia Li-Sien Chong Lee Shin, Zhi Zhao, Guangchang Pei, Han Zhu, Chujin Cao, Xiaofeng He, Yi Huang, Zufu Ma, Liu Liu, Le Wang, Yong Ning, Wei Liu, Gang Xu, Xiaohui Wang, Rui Zeng, Ying Yao

**Affiliations:** 1 Departments of Nephrology, Tongji Hospital, Tongji Medical College, Huazhong University of Science and Technology, 1095 Jiefang Ave, Wuhan, 430030 Hubei China; 2 Departments of Nutrition, Tongji Hospital, Tongji Medical College, Huazhong University of Science and Technology, 1095 Jiefang Ave, Wuhan, 430030 Hubei China; 3 Department of Nephrology, Fifth Hospital in Wuhan, 122 Xianzheng Ave, Wuhan, 430050 Hubei China

**Keywords:** Putative endothelial progenitor cells, Microvesicles, Renal fibrosis, Pericyte–myofibroblast transition

## Abstract

**Background:**

Putative endothelial progenitor cells (pEPCs) have been confirmed to participate in alleviation of renal fibrosis in several ischaemic diseases. However, their mechanistic effect on renal fibrosis, which is characterized by vascular regression and further rarefaction-related pathology, remains unknown.

**Methods:**

To explore the effect and molecular mechanisms by which pEPCs act on unilateral ureteral obstruction (UUO)-induced renal fibrosis, we isolated pEPCs from murine bone marrow. In vivo, pEPCs (2 × 10^5^ cells/day) and pEPC-MVs (microvesicles) were injected into UUO mice via the tail vein. In vitro, pEPCs were co-cultured with renal-derived pericytes. Pericyte-myofibroblast transition was evaluated using the myofibroblast marker α-smooth muscle actin (α-SMA) and pericyte marker platelet-derived growth factor receptor β (PDGFR-β).

**Results:**

Exogenous supply of bone marrow-derived pEPCs attenuated renal fibrosis by decreasing pericyte-myofibroblast transition without significant vascular repair in the UUO model. Our results indicated that pEPCs regulated pericytes and their transition into myofibroblasts via pEPC-MVs. Co-culture of pericytes with pEPCs in vitro suggested that pEPCs inhibit transforming growth factor-β (TGF-β)-induced pericyte–myofibroblast transition via a paracrine pathway.

**Conclusion:**

pEPCs effectively attenuated UUO-induced renal fibrosis by inhibiting pericyte–myofibroblast transition via a paracrine pathway, without promoting vascular repair.

**Electronic supplementary material:**

The online version of this article (10.1186/s13287-019-1201-5) contains supplementary material, which is available to authorized users.

## Background

Fibrosis is the final common pathological morphological change in all forms of chronic kidney disease, characterized by myofibroblast produced massive extracellular matrix deposition in interstitium [1–3]. Unilateral ureteral obstruction (UUO) is a well-established animal model of renal fibrosis caused by post-renal obstruction. Continuous urinary retention increases renal pressure, leading to decreased renal blood flow, thereby exacerbating renal injury, which eventually progresses to irreversible renal fibrosis [4, 5].

Microvessels are consisted of inner lined endothelial cells and outer attached perivascular pericytes, which play an important role in vascular stabilization and permeability. Both direct injury to endothelial cells and the detachment of pericyte from endothelial cell lead to vascular damage and rarefaction. Decrease blood flow in UUO leads to microvascular rarefaction, participating in the initiation and progression of renal interstitial fibrosis [6]. Furthermore, a study demonstrated that pericyte could be a major source of myofibroblast after migrating from microvascular wall. Generation of transgenic collagen 1α1-green fluorescent protein (coll-GFP) mice indicated that a population of pericytes was one of the sources of collagen-producing myofibroblasts in UUO in kidney [7, 8]. While current therapeutic options are centred around improving renal blood flow and decreasing the accumulation of toxins, finding a way to directly alleviate microvascular rarefaction and improve vascular stabilization would provide a more effective method of slowing down the progression of renal fibrosis. Putative endothelial progenitor cells (pEPCs) are a type of haematopoietic stem cell that have a protective effect through their capacity to promote vascular repair [9, 10]. When organs suffer from ischaemic injury or endothelial damage, pEPCs mobilize from the bone marrow and home to injury sites to induce vascular repair, including vascular proliferation and remodelling[9, 11]. Inhibition of pEPC mobilization significantly weakens their protective effect. Our previous study using AMD3100, a small molecule inhibitor of CXCR4, to block the mobilization of pEPCs to injured kidneys in a UUO model confirmed a reduced therapeutic effect of pEPCs on renal fibrosis [12]. Researchers believe that pEPCs protect against organ fibrosis by promoting vascular repair. Some studies have reported that pEPCs regulate vascularization-related factors, such as vascular endothelial growth factor, to regulate vascular endothelial cell interconnection and tube formation [13–16], while others have confirmed that pEPCs can directly differentiate into endothelial cells to promote angiogenesis and vascularization [17, 18]. However, Sradnick and colleagues reported that extrarenal progenitor cells do not contribute to renal endothelial repair directly [19]. Studies have proposed that stem cell-derived microvesicles (MVs) (vesicles approximately 200 nm in diameter) are complex particles formed by exocytosis of the cell membrane of pEPCs and contain a selection of proteins and RNA that can act as messengers to deliver information to distant target cells [20]. Given their characteristic ability to dock to the plasma membrane or be assimilated by endocytosis, pEPC-MVs can be an effective medium for transmission of paracrine signals between cells [21, 22].

In the present study, we investigated the effects of pEPCs on vascular repair and renal fibrosis and further discussed how their effects are mediated. We isolated and cultured pEPCs from murine bone marrow. In vivo, pEPCs were injected into UUO mice via the tail vein to observe the effect of pEPCs on UUO-induced vascular rarefaction and subsequent fibrosis. Our results demonstrated a protective effect of pEPCs on UUO-induced renal fibrosis; however, the protective effect was not due to the widely accepted mechanism of improvement of microvascular rarefaction but rather to the inhibition of pericyte detachment from endothelial cells and the pericyte–myofibroblast transition. Furthermore, our results suggest that the effect of pEPCs on pericyte–myofibroblast transition was dependent on pEPC-MVs.

## Methods and materials

### UUO and ischaemia reperfusion injury (IRI) surgery

All experiments were approved by the Animal Care and Use Committee of Tongji Hospital. Male C57BL/6 mice were purchased from Beijing Huafukang Laboratory Animal Technology Co., Ltd., Beijing, China. The UUO surgery was performed as previously reported [12]. The mice were anesthetized with 1% sodium pentobarbital (8 μl/g, promoter, Wuhan, China). Then, the left ureter was isolated from the surrounding tissues and double ligated. Next, the ureter between the two ligations was cut. At last, 200 ul 0.9% sterile normal saline was injected into the abdominal cavity and the incision was closed under aseptic condition. Mice were divided into three groups: normal; UUO+PBS (UUO and same dose of PBS injection) and UUO+pEPCs/UUO+pEPC-MVs (UUO and pEPCs or pEPC-MVs injection), *n* = 5–8/group. For IRI surgery, the left renal artery of mice was clamped for 30 min (Roboz Surgical Instrument Co, Germany). Throughout the surgeries, animal body temperature was maintained at 36.6–37.2°C with a temperature control machine (FHC, USA). Animals were divided into three groups: normal; IRI and IRI+pEPCs, *n* = 5/group. Animals were sacrificed on UUO-day 5, 7, or 14 and IRI-day 5. The operated left kidneys of the mice were harvested for further research. Each animal experiments were repeated three times.

### Isolation, culture and characterization of bone marrow-derived pEPCs from mice

pEPCs and GFP-pEPCs were isolated from the bone marrow of 4-week-old wild-type and CAG-enhanced green fluorescent protein (EGFP) male mice (JAX #006567, Jackson Laboratory, USA). Femurs and tibias were flushed with phosphate-buffered saline (PBS) (pH 7.4). Cells were collected and filtered through a 70-μm pore filter to clear away bone scraps. Then, the red blood cells were lysed by incubation in a lytic solution for 5 min, and the remaining cells were collected as bone marrow mononuclear cells (MNCs). MNCs were finally resuspended in EGM-2 (Lonza, USA) and seeded on fibronectin (50 μg/mL, BD biocoat)-coated six-well plates and incubated in a 5% CO_2_ incubator at 37 °C. Culture medium was changed every other day. After 7 days, the cells were collected and used as pEPCs for further research. Characterization was performed according to the method described by Xiaozhen Dai [23]. For immunofluorescence staining, cells were seeded on fibronectin-coated six-well culture plates and incubated with acetylated Dil lipoprotein from human plasma (Dil-Ac-LDL, Thermo Fisher Scientific, Waltham, MA, USA) at 37 °C for 4 h, and cells were then washed with PBS twice and incubated with fluorescein isothiocyanate-labelled *Ulex europaeus* agglutinin-1 (FITC-UEA-1, Sigma-Aldrich, St. Louis, MO, USA) at room temperature (RT) for 1 h. For flow cytometry, cells were first incubated with 5% bovine serum albumin (BSA; Sigma) for 15 min and then incubated with anti-mouse CD34-eFluor 660, CD45-FITC, c-kit-FITC, CD14-PE, CD31-PE, CD133-PE, CD309-PE and CD105-PE antibodies at room temperature for 1 h and analyzed with BD fluorescence-activated cell sorting (FACS) flow cytometer. The antibodies used for flow cytometry are listed in Table 1.

**Table 1:** Antibodies used for flow cytometry

Antibody	Company	Product code
eFluro660-CD34	eBioScience	4273958
FITC-CD45	eBioScience	4277449
FITC-c-Kit	BD	7187544
PE-CD14	BD	5286606
PE-CD133	eBioScience	E01480-1393
PE-CD309	BD	555308
PE-Cy7-CD105	eBioScience	4307154
PE-CD31	BD	3151933

### Treatment of UUO mice with pEPCs and pEPC-MVs

A total of 2 × 10^5^ pEPCs or pEPC-MVs collected from the same density of pEPCs were injected into mice through the caudal vein at the first 24 h, 48 h and 72 h after UUO surgery in UUO+pEPCs group. Control group mice were injected with the same dose of PBS (UUO+PBS). The experiment of pEPCs and pEPC-MVs were conducted at different time.

### Histological, immunocytochemical and immunofluorescence staining

The left kidney from each mouse was fixed in 4% paraformaldehyde and embedded in paraffin. The paraffin-embedded kidneys were cut into 3-μm-thick sections and stained with periodic acid Schiff (PAS), Masson trichrome (Masson) and Sirius red staining were used to evaluate renal tubular injury grade and interstitial fibrosis. Tubular damage score evaluation: at least 10 high power field of view in each kidney were examined. Tubular damage included tubular dilation, tubular cell changed from cuboid to flat shape (1 score); brush border injury (1 score), loss (2 score); cast formation (2 score); tubular cell death and detachment (1 score) [24]. For immunocytochemical (IHC) staining, renal sections were stained with antibodies against α-smooth muscle actin (α-SMA; 1:100, Abcam), platelet-derived growth factor receptor β (PDGFR-β; 1:100, Abcam), collagen IV (1:100, Abcam) and phosphorylated-H3 (PH3, 1:200, Abcam) at 4 °C overnight and HRP-conjugated secondary antibodies. For immunofluorescence (IF) staining, sections were stained with primary antibodies against PCNA (1:100, CST), CD31 (1:100, BD), α-SMA (1:50, Abcam), PDGFR-β (1:50, Abcam), Collagen IV (1:100, Abcam), CD34 (1:100, Abcam) and GFP (1; 100, Abcam) at 4 °C overnight and fluorescence-labelled secondary antibodies. Nuclei were stained with 4′6-diamidino-2-phenylindole (DAPI). Staining was carefully quantified in eight to ten randomly captured fields on each slide, and the data were analyzed using Image-Pro Plus software (Media Cybemetics, Rockville, MD, USA) in a blinded manner.

### Western blot analysis

Renal and cell samples were lysed in RIPA lysis buffer (Promoter, Wuhan, China) containing protease inhibitor (Promoter, Wuhan, China). Proteins were separated by SDS-PAGE and transferred to PVDF membranes (Millipore, Billerica, MA, USA). The membranes were blocked with 5% skimmed milk for 1 h at 37 °C and then probed with antibodies against α-SMA (1:5000, Abcam), PDGFR-β (1:3000, Abcam), CD31 (1:3000, BD) and GAPDH (1:4000, Abbkine) at 4 °C overnight. The blots were incubated with HRP-conjugated secondary antibodies for 1 h at 37 °C. The signal intensities of target bands were quantified using ImageJ software (NIH, USA).

### Quantitative real-time PCR (qRT-PCR)

Total RNA was extracted using Trizol reagent according to the manufacturer’s instructions (Invitrogen, USA). Reverse transcription was performed according to the standard protocol using the GoScript reverse transcription system (Promega, USA). Quantitative PCR was conducted using SYBR master mix (Qiagen, Germany) on a Roche light 480II system. The mRNA expression levels of several markers were detected via the comparative cycle threshold (Ct) method and normalized to the expression levels of GAPDH. The sequences of primers used for PCR are listed in Table 2.

**Table 2:** Sequences of primers used for PCR

Gene	Primer
GAPDH	5′-TTGATGGCAACAATCTCCAC-3′
	3′-CGTCCCGTAGACAAAATGGT-5′
α-SMA	5′-GTCCCAGACATCAGGGAGTAA-3′
	3′-TCGGATACTTCAGCGTCAGGA-5′
PDGFR-β	5′-AGGAGTGATACCAGCTTTAGTCC-3′
	3′-CCGAGCAGGTCAGAACAAAGG-5′
Collagen I	5′-ATGGATTCCCGTTCGAGTACG-3′
	3′-TCAGCTGGATAGCGACATCG-5′
Fibronectin	5′-GCTCAGCAAATCGTGCAGC-3′
	3′-CTAGGTAGGTCCGTTCCCACT-5′

### CFDA-SE and PKH26 staining

For CFDA-SE (Thermo Fisher, USA) staining, pEPCs were collected and suspended in 1 mL phosphate buffer saline (PBS) at a density of 1 × 10^7^ cells/mL. A 2 μM CFDA-SE working solution was prepared. The cell suspension was incubated in the 2 μM CFDA-SE working solution at 37 °C for 20 min. Cells were suspended in PBS for further use. PKH26 staining of pEPCs was carried out according to the kit protocol (PKH26GL kit, Sigma, USA). PKH26-stained pEPCs were collected for intravenous injection to track pEPCs in vivo.

### Mouse imaging in vivo

We injected CFDA-SE-dyed pEPCs into mice 24 h after UUO. Time points of 0 h, 3 h, 24 h, 48 h, 72 h and 5 days after pEPC injection were chosen to track pEPCs with an in vivo imager. Fluorescein tracking was performed with an imaging system (IVIS Spectrum, Caliper, USA). To further track pEPCs in the kidneys, the kidneys were scanned separately post mortem.

### Culture of kidney primary pericytes

For kidney pericyte isolation, a whole kidney from healthy mouse was diced and incubated with liberase (0.5 mg/mL; Roche Applied Science, Indianapolis, IN, USA) and DNase (100 U/ml; Roche Applied Science) in Hank’s balanced salt solution at 37 °C for 1 h; the suspension was passed through a 40-μm filter. Finally, cells were resuspended in MACS (magnetic cell isolation and cell separation) buffer (Miltenyi Biotec, Germany) and incubated with primary anti-PDGFR-β antibody for 15 min and then with microbeads for 15 min at 4 °C. We adopted a microbeads separation technique to obtain the positive cells, namely, pericytes. Pericytes were cultured in DMEM/F12 (Thermo Fisher, USA) containing 10% FBS, 1% penicillin-streptomycin and 1% ITS (insulin-iron selenium transfer protein) in gelatin-coated six-well plates. The primary cultured cells used in the study were at passage 1 or passage 2.

### Co-culture of pEPCs with pericytes

Pericytes were cultured in the upper portion of transwell chambers (Corning, USA), and pEPCs were placed in the lower section. Recombinant mouse transforming growth factor-β (TGF-β; 10 ng/mL, R&D system, USA) was used to stimulate pericyte transition to mimic renal fibrosis in vivo. Cells were co-cultured for 72 h. Pericytes were collected for western blotting and qRT-PCR analyses.

### Isolation of pEPC-MVs by ultracentrifugation

After 7 days of culture, cell medium was removed and replaced with serum-free EGM-2 for 24 h. Then, the cell culture supernatant was collected and centrifuged at 500×*g* for 10 min and 20,000×*g* for 20 min to clear away dead cells and large cell debris. The final supernatant was collected for ultracentrifugation at 100,000×*g* for 70 min. The exosome pellets were suspended in PBS for a second ultracentrifugation for further purification. Exosome pellets were resuspended in PBS and kept at 4 °C for short-term storage or − 80 °C for long-term storage.

### Microvesicles identified by electron microscopy

Microvesicles were isolated and purified as described above and fixed in 2% PFA (*w*/*v*) in 200 mM phosphate buffer (pH 7.4). Fixed microvesicles were dropped onto a formvar-carbon-coated grid (PolyScience, USA) and left to dry at RT for 20 min. After washing, microvesicles were fixed in 1% glutaraldehyde for 5 min, washed in water and stained with saturated aqueous uranyl oxalate for 5 min. Samples were then embedded in 0.4% *w*/*v* uranyl acetate and 1.8% *w*/*v* methylcellulose and incubated on ice for 10 min. The excess liquid was removed. The grid was dried at RT for 10 min and viewed at 20,000× magnification using an electron microscope (Hitachi H-7000FA, Japan).

### Statistical analyses

All results are presented as the means ± SD, and all experiments were performed at least in triplicate. Statistical differences between two groups were analyzed with an unpaired *t* test or Mann-Whitney *U* test using GraphPad Prism 5.0 software. Statistical significance was set at *p* < 0.05.

## Results

### Characterization of bone marrow-derived pEPCs

**Fig. 1:**
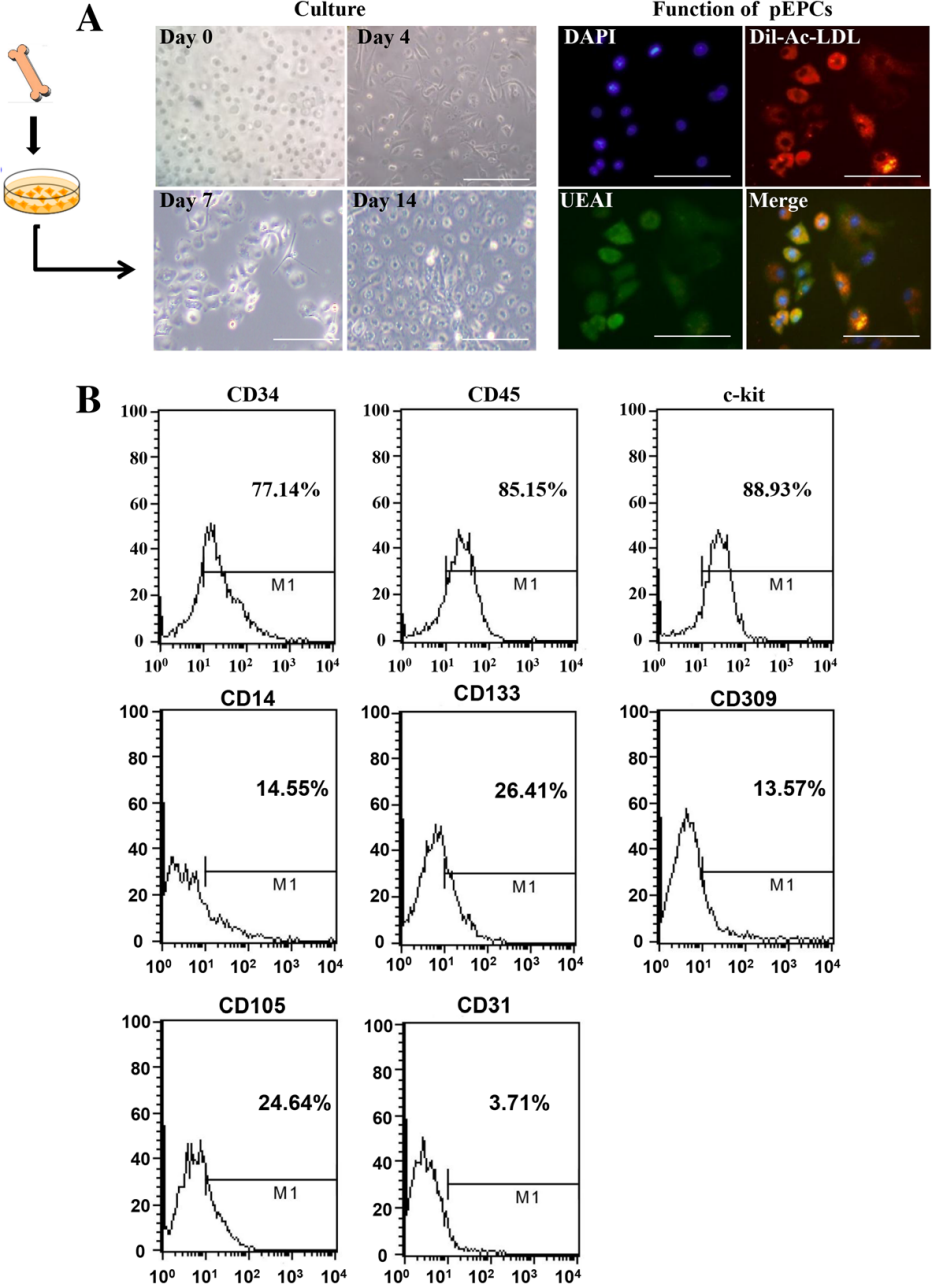
Characterization of bone marrow-derived pEPCs. pEPCs were extracted from murine bone marrow. **a** The morphology of pEPCs after 7 and 14 days of culture was detected with an optical microscope. Early pEPCs showed a spindle-shape morphology on day 7. On day 14, late pEPCs presented an endothelium-like, cobblestone-like morphology. Early pEPCs were positive for Dil-Ac-LDL and UEA-1 (pEPCs incorporated acetylated low-density lipoprotein (red) and bound UEA-I (green)). **b** Flow cytometry showed high expression of CD34, CD45 and c-kit. Meanwhile, early pEPCs were positive for CD14, CD133, CD309 and CD105 and exhibited low CD31 expression. Single samples of 2 × 10^5^ cells were analyzed via flow cytometry, and all the above experiments were carried out at least in triplicate. Scale bar, 200 μm

Exogenous pEPCs were derived from murine bone marrow. At 7 days of culture, early pEPCs became spindle-shaped. At 14 days, the cells presented an endothelium-like, cobblestone-like morphology [23]. Functional assays confirmed that early pEPCs were positive for Dil-Ac-LDL and UEA-1 (pEPCs incorporated acetylated low-density lipoprotein (red) and bound UEA-I (green); Fig. 1a). Next, we identified pEPCs via flow cytometry. Single samples of 2 × 10^5^ cells were analyzed, and all the above experiments were carried out at least in triplicate (Fig. 1b). Flow cytometry showed that early pEPCs had high CD34 expression (77.14%), a marker found on early haematopoietic and vascular-associated tissue [25, 26]. As bone marrow-derived cells, pEPCs presented high expression of the typical myeloid marker CD45 (85.15%) and the stem cell marker c-kit (88.93%). CD14 is a monocyte lineage marker, and we detected lower CD14 expression (14.5%) on bone marrow-derived pEPCs compared to monocyte-derived pEPCs [27]. CD133 and CD105 are two markers represent the proliferative capacity of pEPCs. During the culture of pEPCs, the expressions of CD133 and CD105 reduced, meaning a differentiation capacity into endothelial cells [25, 28]. CD309, also called vascular endothelial growth factor receptor-2, is mainly expressed on endothelial cells and is an early marker of pEPCs. CD31 is a marker of mature endothelial cell. pEPCs as endothelial precursor had only 3.71% CD31 expression during the early stage of pEPCs culture [23]. These characteristics were in accordance with previous descriptions of pEPCs [29–31].

### Exogenous pEPC injection ameliorated renal fibrosis

**Fig. 2:**
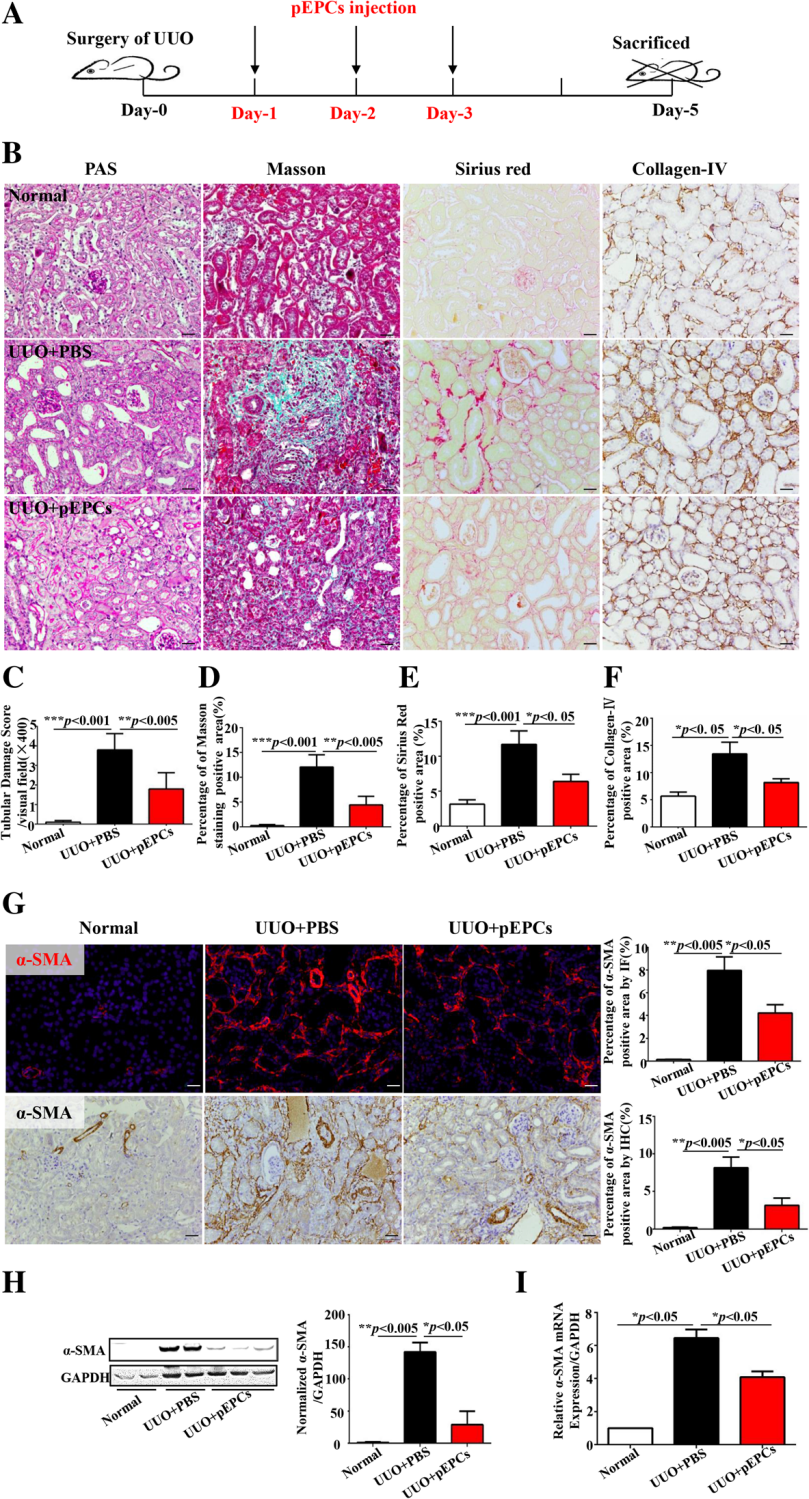
Exogenous pEPC injection ameliorated renal fibrosis. **a** Experimental scheme: UUO day 5 kidneys were harvested. pEPCs were injected at 24-, 48- and 72-h post-surgery. **b**–**f** PAS staining was used to observe tubular morphological changes: tubular brush border, dilation and cast formation. Masson trichrome (green) and Sirius red (red) staining and IHC staining for Collagen IV (brown) were used to evaluate extracellular matrix deposition in the renal interstitium. **g** IHC and IF to detect α-SMA was employed to identify myofibroblasts after UUO. **h**, **i** Western blotting and qRT-PCR were performed to measure α-SMA protein and RNA expression levels. The data are presented as the mean ± SD; *n* = 5/group. *p < 0.05; **p < 0.005; ***p < 0.001. Scale bar, 20 μm

We administered intravenous injections at 24-, 48- and 72-h post-surgery to exogenously supply mice with pEPCs, and at day 5 after UUO, kidneys were harvested (Fig. 2a). We detected an improvement in renal tubular injury and fibrosis scores after injection of pEPCs (UUO+pEPCs) (Fig. 2b–f). PAS staining revealed ameliorated pathological changes, including epithelial cell brush border loss, tubular dilation and cast formation, in the UUO+pEPCs group compared with the UUO+PBS group. Masson trichrome (Masson) and Sirius red staining further showed an approximately 50% decrease in interstitial collagen deposition in the UUO+pEPCs group. IHC for Collagen IV also showed sparser extracellular collagen deposition after treatment with pEPCs. We further explored the effects of pEPCs on myofibroblasts by evaluating α-SMA staining. IHC and IF results showed widespread α-SMA expression in the renal interstitium after UUO (a nearly eightfold increase compared with the normal group evaluated by both IHC and IF). α-SMA in the renal interstitium was reduced by approximately 50% after pEPC injection (Fig. 2g). Western blotting and quantitative real-time PCR showed a consistent effect of pEPCs on α-SMA expression (Fig. 2h, i). In summary, pEPC injection alleviated UUO-induced tubular injury and renal fibrosis.

### pEPCs did not improve UUO-induced capillary rarefaction

**Fig. 3:**
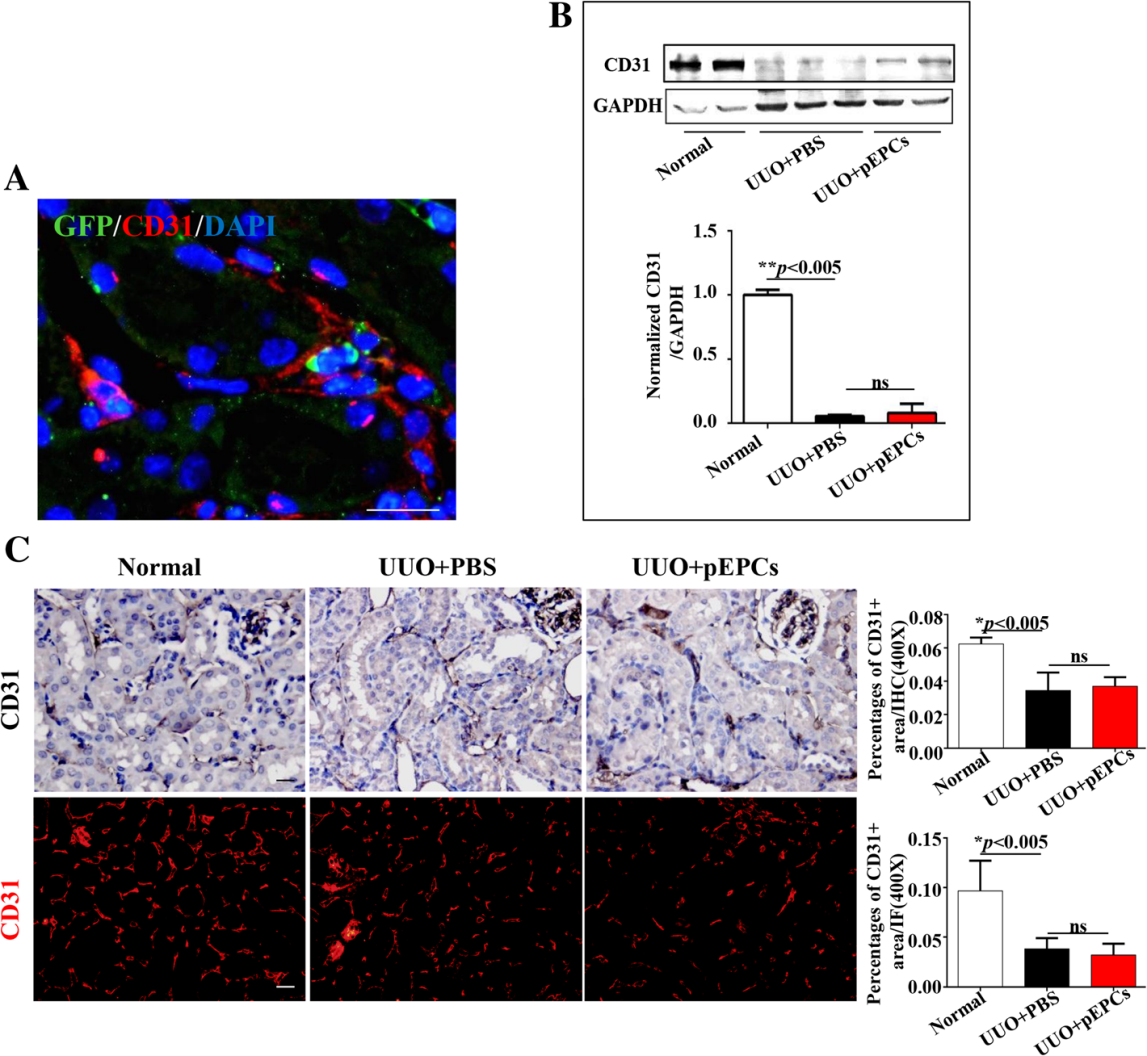
pEPCs did not improve UUO-induced capillary rarefaction. **a** GFP-pEPCs tracking (green) showed exogenous pEPCs located in the renal interstitium in the cortex and the cortico-medullary junction, surrounded by CD31-positive (red) endothelial cells. **b**, **c** Western blotting, IHC and IF showed that UUO led to significant endothelial cell damage, and pEPC treatment did not improve capillary rarefaction. The data are presented as the mean ± SD; *n* = 5/group. **p < 0.005; ns, no significant difference. Scale bar, 20 μm

We demonstrated a therapeutic effect of pEPC injection on UUO-induced renal injury and fibrosis. To explore whether this protective effect was due to its most widely recognized function in vascular repair, we tested CD31, a marker present in mature endothelial cells. We injected pEPCs isolated from GFP-positive transgenic mice into UUO mice. The results showed that GFP-pEPCs from CAG-EGFP male mice were located in the renal interstitium, surrounded by CD31-positive endothelial cells (Fig. 3a), which indicated that the injected pEPCs homed to the injured kidney and interacted with endothelial cells. However, the western blotting results showed no significant increase in CD31-positive endothelial cells in the UUO+pEPCs group compared with the UUO+PBS group (Fig. 3b), hinting that pEPCs had no effect on UUO-induced capillary rarefaction. IHC and IF for CD31 further supported the western blotting results (Fig. 3c). We observed that pEPCs homed to the site of injury but vascular rarefaction was not improved, which was similar to the result of a 2016 study by Sradnick [19]. Thus, we were curious about how pEPCs attenuated renal injury and fibrosis apart from a direct interaction with endothelial cells. We considered whether exogenous pEPCs acted on other cell types, such as tubular epithelial cells, pericytes or fibroblasts, through other pathways.

### pEPC tracking

**Fig. 4:**
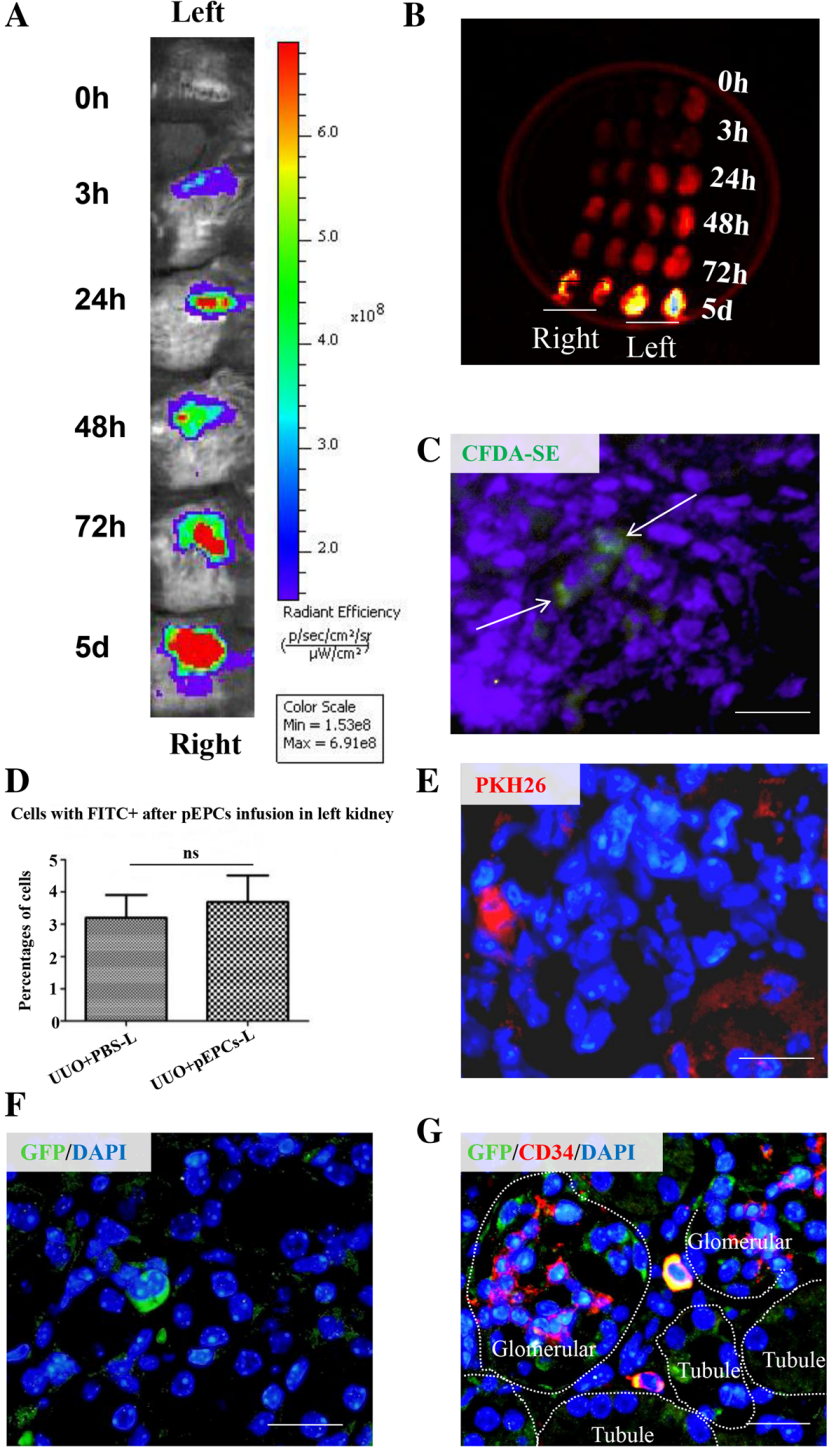
pEPCs tracking. **a** pEPCs were dyed with CFDA-SE and injected into mice 24 h after UUO surgery. Time points of 0 h, 3 h, 24 h, 48 h, 72 h and 5 days after pEPC injection were chosen to track the fluorescein intensity. An increased fluorescence intensity (red represents the strongest fluorescence intensity) was observed over time after surgery. **b** Both the operated left kidneys and the non-operated right kidneys were harvested and scanned for fluorescence intensity to eliminate errors. Kidneys presented a much stronger fluorescence on UUO day 5 than at other time points. **c**, **e** and **f** Mice were injected with PKH26 and CFDA-SE-dyed pEPCs and GFP-labelled pEPCs. Sparse pEPCs were found in frozen renal sections or paraffin-embedded renal sections. **d** Flow cytometry for CFDA-SE showed no significant difference between the operated left kidneys with (UUO+pEPCs-L group) or without pEPCs (UUO+PBS group). **g** IF staining for GFP (green) and the pEPC marker CD34 (red) showed a phenomenon consistent with PKH26 and CFDA-SE tracking: only sparse GFP and CD34-positive cells were detected in left kidney. All the data are presented as the mean ± SD; *n* = 2–3/group, and 5 to 6 kidney sections were analyzed. ns, no significant difference. Scale bar, 20 μm

To explore the pathways by which exogenous pEPCs participated in renal repair and protection, we tagged pEPCs with different dyes for tracking via an in vivo imager, IF and flow cytometry. At first, we injected CFDA-SE-dyed pEPCs into mice 24 h after UUO. Time points of 0 h, 3 h, 24 h, 48 h, 72 h and 5 days after pEPC injection were chosen to track the fluorescein intensity. We detected increased fluorescence intensity accumulation over time, with the highest fluorescence intensity observed on day 5 (Fig. 4a). Further, we harvested both the operated left kidneys and the control right kidneys for simultaneous assessment of fluorescence intensity. A similar trend over time was observed, both the operated (left) and control (right) kidneys presented fluorescence congregation, but the operated kidney displayed stronger intensity. The fluorescence accumulation increased as injury progressed and peaked on day 5 (Fig. 4b). To further track pEPC numbers in operated kidneys, we injected mice with PKH26- and CFDA-SE-dyed pEPCs and harvested operated kidneys on day 5. However, only few positive cells were found in frozen renal sections and in paraffin-embedded renal sections (Fig. 4c, e). Next, CFDA-SE-labelled pEPCs were measured using flow cytometry at 3 h after pEPCs treatment. No significant difference was observed between the control group (UUO+PBS-L) and pEPC injection group (UUO+pEPC-L) (Fig. 4d). Furthermore, we isolated GFP-pEPCs from CAG-EGFP mice, which offered the advantage of endogenous fluorescence for more efficient tracking. The GFP+ cells were observed in the renal interstitium in the cortex and the cortico-medullary junction. IF co-staining for GFP and CD34, a marker representing pEPCs, showed a phenomenon consistent with the PKH26 and CFDA-SE labelling results: the tracked GFP-positive cells were CD34-positive pEPCs (Fig. 4f and g). The inconsistency between the sparsity of tracked cells and the acquired strong fluorescence intensity at the injured site could be explained by the possibility that only a few pEPCs homed to the injured site, while the strong fluorescence intensity might originate from factors encased in the dyed plasma membrane that were transported via pEPC-derived MVs. We inferred that pEPC-MVs reached the injured site through circulating blood and delivered their signalling factors to recipient cells in the kidney, such as endothelial cells and pericytes tightly attached to endothelial cells.

### pEPCs regulated migration of pericytes away from endothelial cells and reduced pericyte–myofibroblast transition

**Fig. 5:**
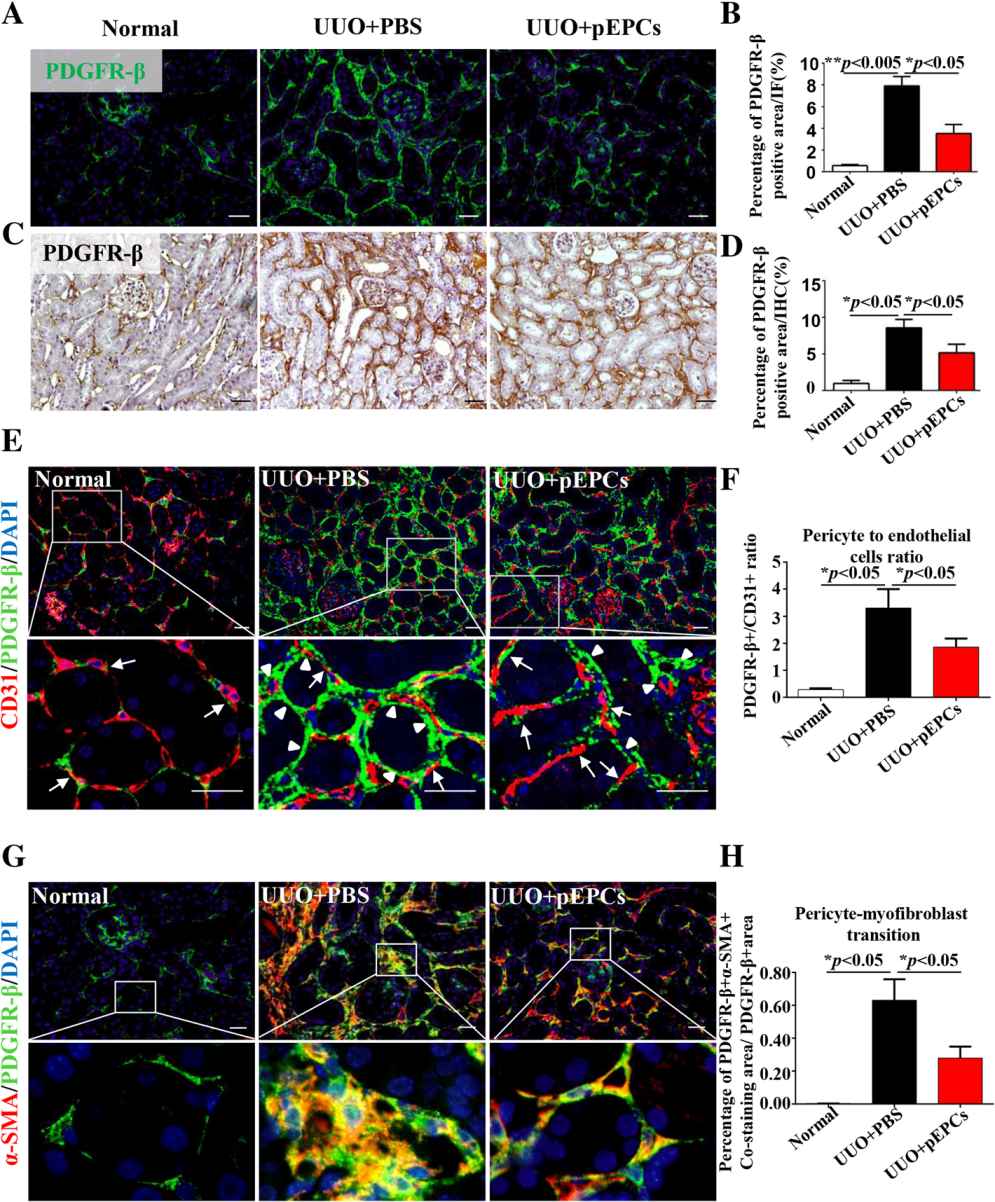
pEPCs regulated migration of pericytes away from endothelial cells and reduced pericyte–myofibroblast transition. **a**, **c** IF and IHC demonstrated a significant increase in the number of pericytes in the renal interstitium after UUO. pEPC infusion reduced the expression of pericytes. **b**, **d** The histogram demonstrates a nearly 50% reduction in PDGFR-β-positive pericytes in the UUO+pEPCs group after pEPC treatment. **e** Further double IF staining for CD31 (red) and PDGFR-β (green) revealed that pericytes were located at a farther distance from endothelial cells in the UUO+PBS group. pEPCs promoted pericyte attachment to endothelial cells and inhibited pericyte proliferation in the interstitium (arrows represent normal attachment of pericyte to endothelial cells, while triangles represent migration of pericytes away from endothelial cells). **f** Statistical analysis of pericyte to endothelial cell ratio. In a normal kidney, the pericyte to endothelial cell ratio was small. In the UUO+PBS group, increased pericyte and decreased endothelial cell led to a ratio of 3.31:1. In the UUO+pEPCs group, the ratio decreased. **g**, **h** Co-staining for α-SMA (red) and PDGFR-β (green) showed extensive pericyte–myofibroblast transition (yellow) in the renal interstitium after UUO. In the UUO+pEPCs group, the co-expression of α-SMA and PDGFR-β/PDGFR-β positive area became less pronounced. All the data are presented as the mean ± SD; *n* = 3–4/group. *p < 0.05; **p < 0.005. Scale bar, 20 μm

Structurally, pericytes and endothelial cells share a common basement membrane and gap junctions, which allow for communication and further sustain the vascular structural stability [32]. Endothelial cell damage leads to pericyte detachment from endothelial cells, migration into the interstitium and subsequent proliferation and transition. In 2008, pericytes were identified as the primary source of interstitial myofibroblasts in the fibrotic kidney. PDGFR-β has already been verified as a pericyte marker and was therefore chosen to label pericytes [7]. As shown in Additional file 1: Figure S1, on UUO days 7 and 14, co-staining of PDGFR-β and CD31 showed that the space between pericytes and endothelial cells (white arrow, white triangles) increased, indicating that pericytes separated from endothelial cells. Detached pericytes in interstium showed a proliferative ability by co-staining of proliferating cell nuclear antigen (PCNA) (red triangle) and PDGFR-β (green). Pericyte proliferation speed peaked approximately 48 h after UUO and then gradually slowed (data not shown). However, the number of pericyte kept increased from UUO day 7 to 14 with a higher density expression of PDGFR-β in UUO day14. Otherwise, co-staining of α-SMA (marker of myofibroblast) and PDGFR-β (yellow areas) showed that the transition of pericyte into myofibroblast increased too. Pericyte-transited myofibroblasts were evenly distributed around renal tubules on day 7 but accumulated into denser areas in the interstitium by day 14. pEPC injection significantly decreased the presence of pericytes (Fig. 5a–d). Next, co-staining of PDGFR-β and CD31 was used to observe the location changes of pericytes relative to endothelial cells. As shown in Fig. 5e, in the normal group, pericytes showed a characteristic narrow fibrillar morphology and were closely attached to the outside of endothelial cells (white arrow). In the UUO+PBS group; although some pericytes showed a conserved normal morphology and relationship with endothelial cells, more pericytes moved away from nearby endothelial cells and became thick (white triangle). The pericyte to endothelial cell ratio varied from 0.28:1 in the normal group to 3.31:1 in the UUO+PBS group. In the UUO+pEPCs group, the ratio decreased to 1.86:1, indicating a decreased pericyte expression after pEPCs treatment (Fig. 5f). pEPC injection reduced these pathological structural changes between pericytes and endothelial cells. Furthermore, we examined α-SMA and PDGFR-β co-expression via immunofluorescence and found that pEPCs reduced pericyte–myofibroblast transition after UUO (Fig. 5g, h). From the above data, we concluded that pEPCs alleviate renal fibrosis by decreasing pericyte migration and transition into myofibroblasts.

### pEPCs lessened UUO-induced renal epithelial cell G2/M arrest

**Fig. 6:**
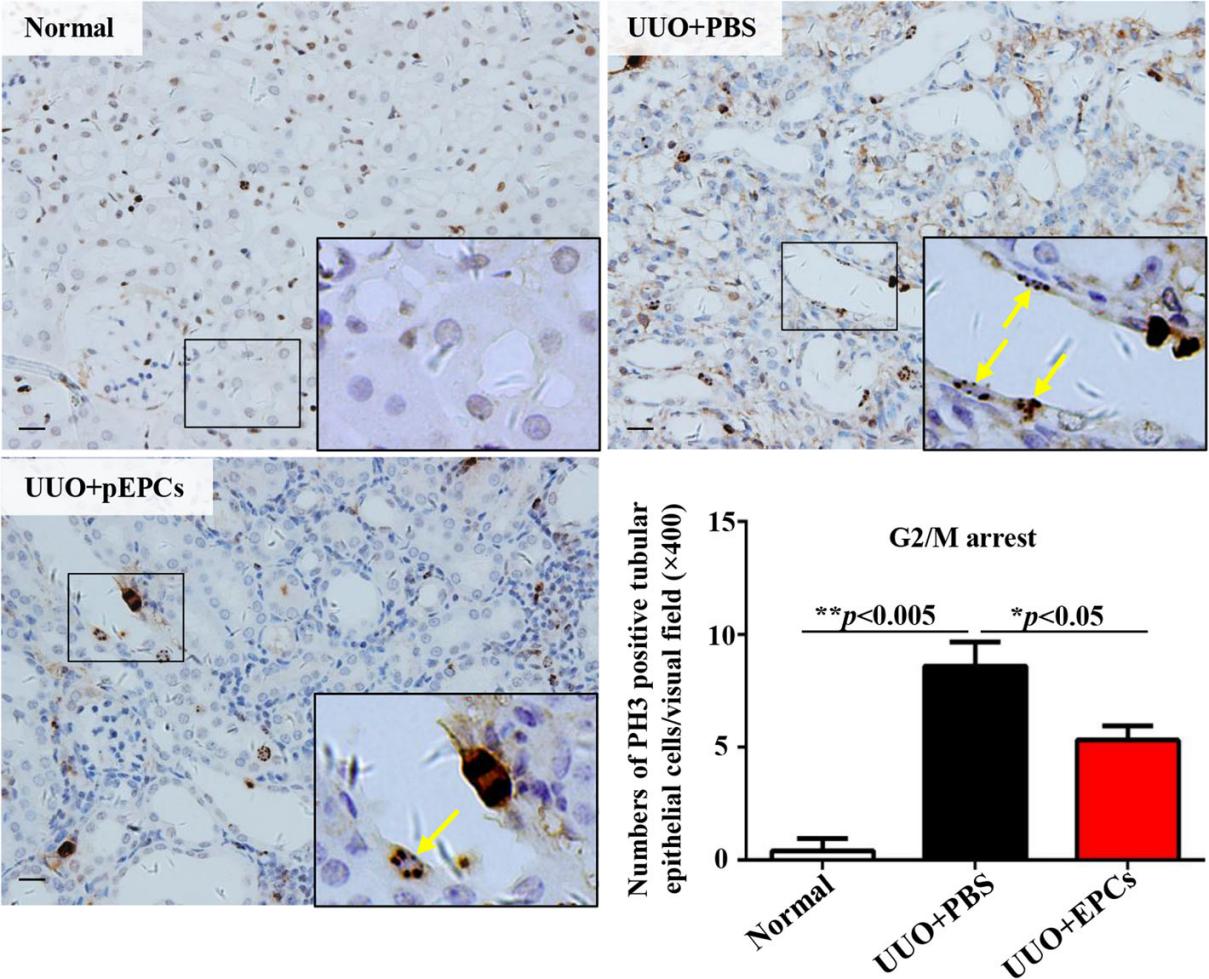
pEPCs lessened UUO-induced renal epithelial cells arrested in G2/M. Increased PH3-positive renal tubular epithelial cells (yellow arrow: the brown cell with dotted nucleus) were found in UUO-induced kidney injury. In the UUO+pEPCs group, the number of G2/M-arrested cells decreased. Data are counted as the mean ± SD, *n* = 5/group. *p < 0.05; **p < 0.005. Scale bar, 20 μm

In addition to regulating pericytes, the effects of pEPCs on tubular epithelial cells (TECs) were also observed. PH3 immunohistochemistry was performed to investigate G2/M arrest after UUO. After UUO, TECs arrested in G2/M were observed. pEPC injection decreased the number of G2/M-arrested TECs (Fig. 6). G2/M cell cycle arrest of TECs was demonstrated to have a causal relationship with renal fibrosis after injury (G2/M) [33]. As pEPCs were administered via vein injection, pEPCs were at first directly communicated with vascular endothelial cells. Based on special gap junction between pericyte and endothelial cells, we guessed that pEPCs might lead to production of some factors by endothelial cells, which were transferred through gap junction to indirectly regulate pericytes. However, given the lack of specific channels or interactions between pEPCs and TECs, we became interested in how pEPCs indirectly regulated TECs.

### pEPC-MVs reduced pericyte–myofibroblast transition in vivo

**Fig. 7:**
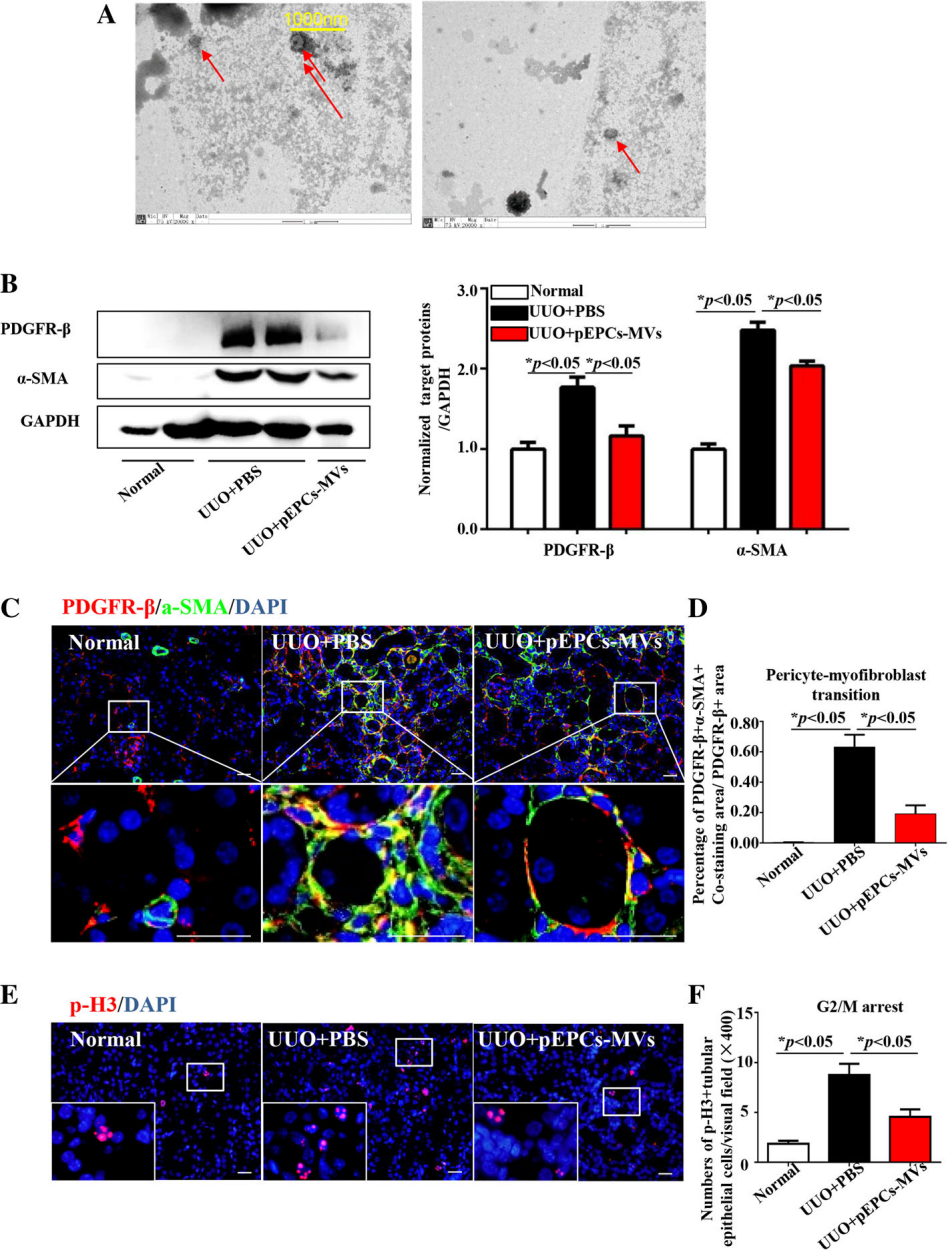
pEPC-MVs reduced pericyte–myofibroblast transition in vivo. **a** Representative electron microscopic image showing pEPC-MVs (red arrows) to be approximately 200 nm in diameter. **b** pEPCs-MV injection significantly reduced α-SMA and PDGFR-β protein expression levels after UUO surgery. **c**, **d** Co-staining for α-SMA (green) and PDGFR-β (red) showed that UUO led to extensive pericyte–myofibroblast transition (yellow) in the renal interstitium. After pEPCs-MV treatment, the co-expression of α-SMA and PDGFR-β became sparser. **e**, **f** G2/M arrest in epithelial cells was evaluated by IF staining for PH3 (red), which showed that the number of TECs at G2/M arrest decreased after treatment with pEPC-MVs. The data are presented as the mean ± SD; *n* = 3–4/group. *p < 0.05

Based on our above results, we extracted pEPC-derived MVs (pEPC-MVs) through ultracentrifugation. MVs are in diameter range from 100 to 1000 nm and generated by blebbing of the plasma membrane to be secreted by exocytosis [21]. Thus, MVs contain both partial plasma membrane and intracellular contents to introduce MVs in details. We observed approximately 200 nm in diameter MVs with clear plasma membrane (Fig. 7a) by electron microscopy imaging. In vivo experiment injection of pEPC-MVs led to a significant reduction in α-SMA and PDGFR-β protein expression levels after UUO surgery, concurrent with alleviation of renal fibrosis (Fig. 7b). Further evaluation of α-SMA and PDGFR-β co-staining showed decreased pericyte–myofibroblast transition after pEPCs-MV treatment (Fig. 7c, d), demonstrating that pEPCs could affect pericytes through MVs. A reduction in PH3 positive-TECs in the UUO+pEPC-MVs group (Fig. 7e, f) further demonstrated that pEPCs affected TECs through the action of MVs. Thus, we concluded that pEPCs alleviate renal fibrosis by regulating pericytes and pericyte–myofibroblast transition, as well as TECs, via MVs.

### pEPCs reduced pericyte–myofibroblast transition via a paracrine effect in vitro

**Fig. 8:**
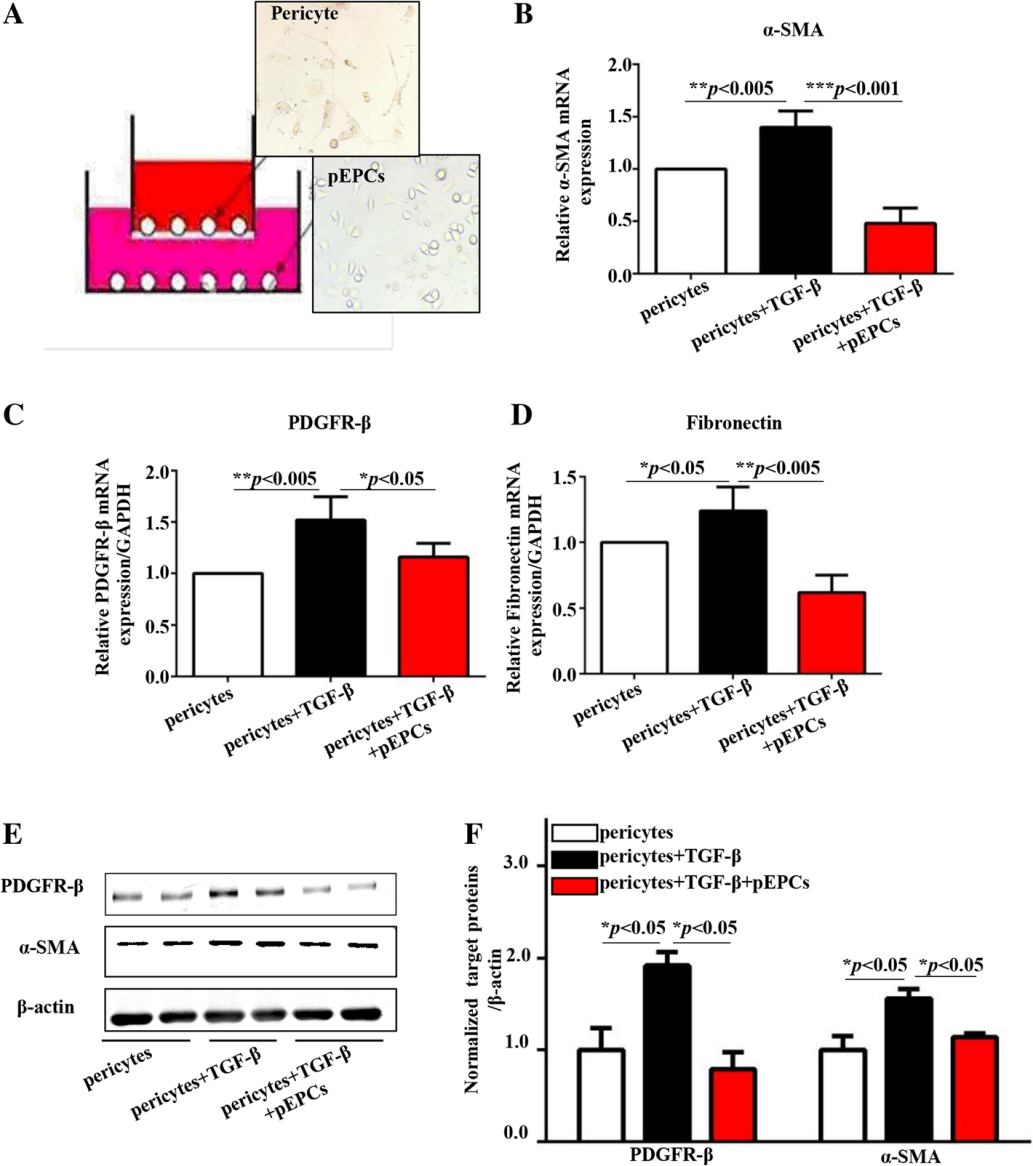
pEPCs reduced pericyte–myofibroblast transition via a paracrine effect in vitro. **a** Pericytes were co-cultured with pEPCs in transwell chambers. Then, 10 ng/ml TGF-β was used to stimulate pericyte transition into myofibroblasts. In the pericyte and pericytes+TGF-β groups, pEPCs were replaced with EBM-2 culture medium. **b**–**d** TGF-β increased the mRNA expression of α-SMA, PDGFR-β and fibronectin, while co-culture with pEPCs attenuated the effect of TGF-β on pericytes. **e**, **f** Western blotting showed similar changes in protein levels representative of pericyte–myofibroblast transition when pericytes were co-cultured with pEPCs. The data are presented as the mean ± SD; *n* = 3/group. *p < 0.05; **p < 0.005; ***p < 0.001

To further confirm that the therapeutic effect of pEPCs in UUO was via a paracrine pathway, we co-cultured pEPCs with pericytes in transwell chambers, which prevented cell pass while allowing the pass of cell-derived factors. TGF-β and its receptor are expressed ubiquitously by most cell types. As TGF-β pathway participates in regulating the expression of fibrotic genes such as collagens and fibronectin, increased level of TGF-β can lead to fibrosis both in vivo and vitro experiments [34–36]. In the presence of TGF-β, pericytes increased and underwent a morphological change through transition into α-SMA-positive myofibroblasts. When co-cultured with pEPCs, pericyte expression of α-SMA decreased (Fig. 8b). The expression of PDGFR-β and the extracellular matrix deposition were also reduced compared with pericytes cultured in EGM-2 medium (Fig. 8c, d). Western blotting showed a similar inhibition of pericyte proliferation and pericyte–myofibroblast transition when pericytes were co-cultured with pEPCs (Fig. 8e, f). These in vitro experiments supported the notion that pEPCs reduce pericyte-fibroblast transition via a paracrine pathway.

### pEPCs alleviated kidney microvascular rarefaction after ischaemia reperfusion injury

To further explore whether our differing results concerning post-renal injury vascularization could be due to the animal model used, we used another renal injury animal model, namely, ischaemia reperfusion injury (IRI) in mice. Immunofluorescence staining for CD31 showed alleviation of vascular rarefaction after injection of pEPCs, which was consistent with previous reports [23, 31] (Additional file 3: Figure S3).

## Discussion

Numerous studies have demonstrated that pEPCs play an important role in vascular proliferation and repair after organ ischaemic or endothelial damage [10, 37, 38]. Some studies have described an anti-fibrotic effect of pEPCs in ischaemic myocardium [39] and chronic liver injury [40] through their capacity for vascular repair. In the present study, we explored the role of pEPCs in a UUO model and found significantly reduced expression of α-SMA and Collagen IV, indicating a protective effect of pEPCs against renal fibrosis. However, the reno-protective effect of pEPCs was independent of its conventional vascular repair function. Instead, pEPCs ameliorated UUO-induced renal fibrosis by regulating pericytes and inhibiting pericyte–myofibroblast transition.

Pericytes derive from FOXD1+ progenitors and participate in vascular homeostasis in the kidney. In the normal state, pericytes share a common basement membrane and communicate with endothelial cells via intercellular junctions, such as connexins and occludins [32, 41, 42], to sustain the microvascular structure. In abnormal states, factors such as platelet-derived growth factor and TGF-β promote pericyte migration, detachment from endothelial cells, proliferation and transition into other cell types, such as FOXD1+ lineage myofibroblasts. Pericyte–myofibroblast transition has been demonstrated to be the origin of myofibroblasts in chronic kidney disease [8, 43]. In our study, the pericyte to endothelial cell ratio varied from 3.31:1 in the UUO+PBS group to 1.86:1 in the UUO+pEPCs group. As the pericyte to endothelial ratio is less than 0.4 in normal kidneys [7, 32], the increased pericytes might be an endogenous origin of “fibroblasts” through proliferation and transition into myofibroblasts, thereby leading to renal fibrosis.

Our tracking experiment uncovered only a few pEPCs despite the strong fluorescein intensity in injured kidneys, which cannot explain the significant effect of pEPCs on pericyte. Otherwise, under the premise that TECs has no direct interaction with vessels or endothelial cells as pericyte, we observed an influence of injected pEPCs on TECs. Thus, pEPCs might regulate pericyte and TEC via an indirectly way. Lots of evidence demonstrated that microRNAs played important roles in regulating EMT and angiogenesis [44–48]; considering that MVs contains proteins and microRNAs [20, 21, 49], we guessed that pEPCs might regulate pericyte and TEC via microRNAs or proteins in pEPC-MVs. Our in vitro and in vivo experiments both supported the hypothesis that pEPCs alleviated UUO-induced renal fibrosis via the action of pEPC-MVs. Recent studies have shown that pEPCs are not only directly involved in angiogenesis by integration into blood vessels but also regulate the entire microenvironment by secretion of microparticles and microRNAs [50].

Despite our results showing the therapeutic effects of pEPCs in renal fibrosis by alleviating pericyte–myofibroblast transition, we were still curious about the insufficient vascular repair capacity of pEPCs observed in this study. We speculated that our divergent finding might be attributed to the uniqueness of the UUO model. After UUO, continuous squeezing of the operated kidney by urine or residual liquid increases the intrarenal pressure drastically enough to decrease renal blood flow and limit vascular repair caused by pEPCs. The significantly increased number of pericytes and myofibroblasts which accumulated in the renal interstitium on UUO days 7 and 14 (Additional file 2: Figure S2) further decreased the space for efficient neovascularization and vascular repair [51]. To demonstrate our hypothesis, we also investigated the IRI model, in which pathological changes are caused by transient ischaemia reperfusion without continuous damage. Our data showed that the microvascular rarefaction after IRI was improved after pEPC injection (Additional file 3: Figure S3), which confirmed our hypothesis that continuous obstructive damage after UUO limited the vascular repair and neovascularization capacity of pEPCs.

The above data indicated that pEPCs alleviated renal fibrosis by reducing pericyte–myofibroblast transition. However, some questions have yet to be answered: (1) the specific molecular signalling pathways by which pEPCs or pEPC-MVs regulate pericyte-endothelial cell attachment remain unclear; (2) more evidence should be provided to verify whether the effect of pEPCs on pericyte–myofibroblast transition is dependent on pericyte regulation, such as by using pericyte specific knock-out mice to improve the integrity of the experiment; and (3) we observed vascular repair in the IRI model, and thus, whether the predominant mechanism of action of pEPCs in alleviating renal fibrosis is regulation of vascular repair or pericyte–myofibroblast transition needs to be explored.

As stem cell transplantation has become a trending topic in clinical therapy in recent years, more attention should be paid to pEPC research. Our study demonstrated therapeutic effects of pEPCs on renal fibrosis through reduction of pericyte–myofibroblast transition via pEPC-MVs. Our following experiment observed that 30 min after pEPCs injection, sparse pEPCs were found in injured kidney; on the contrary, cell debris were found in the liver and spleen (data not shown). This could further demonstrate that pEPCs worked via a paracrine way. pEPC-MVs carried with some effective proteins, RNAs in pEPCs reached recipient cells and transfer these factors via endocytosis by recipient cells. By transferring, some factors participate in cellular matrix degradation, vascular repair and anti-fibrotic process. Further exploration of the detailed molecular and cellular mechanisms by which pEPCs ameliorate fibrosis should be conducted to ensure safer application in clinical settings.

## Conclusions

In the present study, bone marrow-derived pEPCs were injected into mice after UUO-induced renal fibrosis to observe the effect and mechanisms of action of pEPCs in renal fibrosis. We found that pEPCs significantly attenuated renal fibrosis without promoting vascular repair; instead, pEPCs inhibited pericyte–myofibroblast transition and tubular epithelial cell G2/M arrest. Our results indicated that pEPCs attenuate fibrosis via pEPC-MVs. This study offers a promising stem cell-derived therapeutic option for renal fibrosis.

## Additional files

Additional file 1: **Figure S1.** The location, proliferation and transition of pericytes on UUO days 7 and 14. (A) PDGFR-β+CD31 co-staining showed that in the normal group, pericytes presented a characteristic narrow fibrillar morphology and were closely attached to the outside of endothelial cells (white arrow). Distance between CD31+ (red) endothelial cells and PDGFR-β+ (green) pericytes increased (white triangles) over time, revealing a detachment of pericytes from endothelial cells. (A and B) Co-staining for PCNA (red) and PDGFR-β (green) showed UUO-induced proliferation of pericytes. (A and C) Co-staining for α-SMA (red) and PDGFR-β (green) showed the number of α-SMA+PDGFR-β+ cells (yellow)/PDGFR-β+ cells (green) increased from UUO day 7 to day 14, implying a gradual increase in pericyte–myofibroblast transition. Data are counted as mean ± SD, *n* = 4/group. *P < 0.05. Scale bar, 20 μm. (TIF 22160 kb)

Additional file 2: **Figure S2.** PDGFR-β and α-SMA expression increased over time in post-UUO kidneys. (A) IF and IHC staining for PDGFR-β showed an increased number of pericytes over time in the renal interstitium. (B and C) Western blotting and qRT-PCR showed significantly increased expression of PDGFR-β and α-SMA on UUO days 7 and 14. The data are presented as the mean ± SD; *n* = 5/group. *p < 0.05; **p < 0.005. (TIF 19464 kb)

Additional file 3: **Figure S3.** pEPCs alleviated kidney microvascular rarefaction after ischaemia reperfusion injury. Mice were subjected to ischaemia reperfusion injury (IRI) and sacrificed on day 5. CD31 staining (red) showed that endothelial cells decreased on IRI-day 5. Exogenous injection of pEPCs promoted vascular repair and reduced capillary rarefaction. The data are presented as the mean ± SD; *n* = 4–5/group. *p < 0.05. Scale bar, 20 μm. (TIF 8318 kb)

## Abbreviations

EGFP: Enhanced green fluorescent protein
FACS: Fluorescence-activated cell sorting
GFP: Green fluorescent protein
IF: Immunofluorescence
IHC: Immunohistochemistry
IRI: Ischaemia reperfusion injury
MVs: Microvesicles
PAS: Periodic acid Schiff reaction
PDGFR-β: Platelet-derived growth factor receptor β (pericyte marker)
pEPCs: Putative endothelial progenitor cells
RT: Room temperature
TGF-β: Transforming growth factor-β
UUO: Unilateral ureteral obstruction
α-SMA: α-Smooth muscle actin
