# Supplementary figures and images for: Putative endothelial progenitor cells do not promote vascular repair but attenuate pericyte–myofibroblast transition in UUO-induced renal fibrosis
Source: Stem Cell Res Ther. 2019 Mar 21;10:104. doi: 10.1186/s13287-019-1201-5 (PMC6429829; doi:10.1186/s13287-019-1201-5)

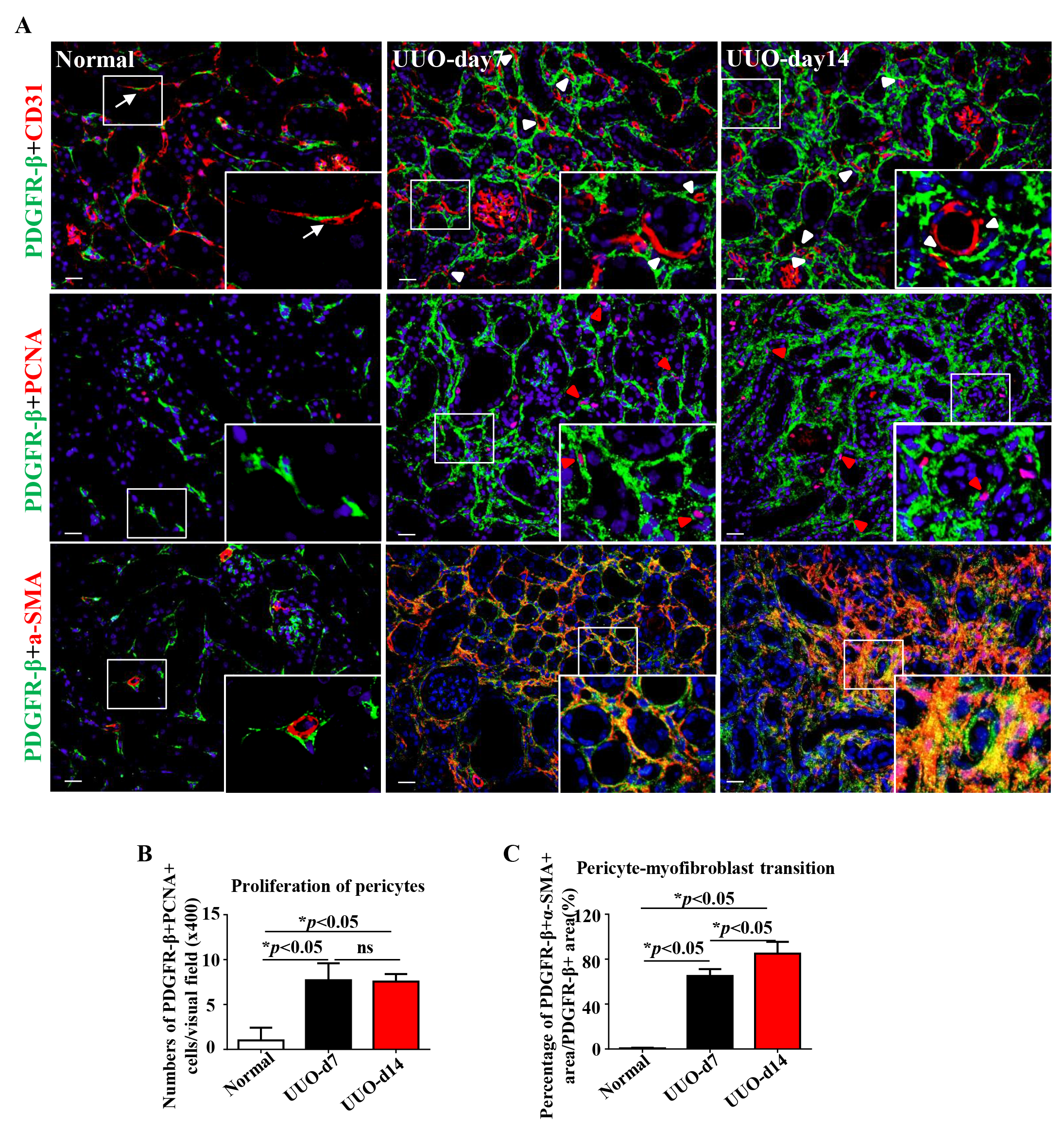

Supplement: Supplementary file 1 — Figure S1. The location, proliferation and transition of pericytes on UUO days 7 and 14. (A) PDGFR-β+CD31 co-staining showed that in the normal group, pericytes presented a characteristic narrow fibrillar morphology and were closely attached to the outside of endothelial cells (white arrow). Distance between CD31+ (red) endothelial cells and PDGFR-β+ (green) pericytes increased (white triangles) over time, revealing a detachment of pericytes from endothelial cells. (A and B) Co-staining for PCNA (red) and PDGFR-β (green) showed UUO-induced proliferation of pericytes. (A and C) Co-staining for α-SMA (red) and PDGFR-β (green) showed the number of α-SMA+PDGFR-β+ cells (yellow)/PDGFR-β+ cells (green) increased from UUO day 7 to day 14, implying a gradual increase in pericyte–myofibroblast transition. Data are counted as mean ± SD, n = 4/group. *P < 0.05. Scale bar, 20 μm. (TIF 22160 kb) [file 13287_2019_1201_MOESM1_ESM.tif]

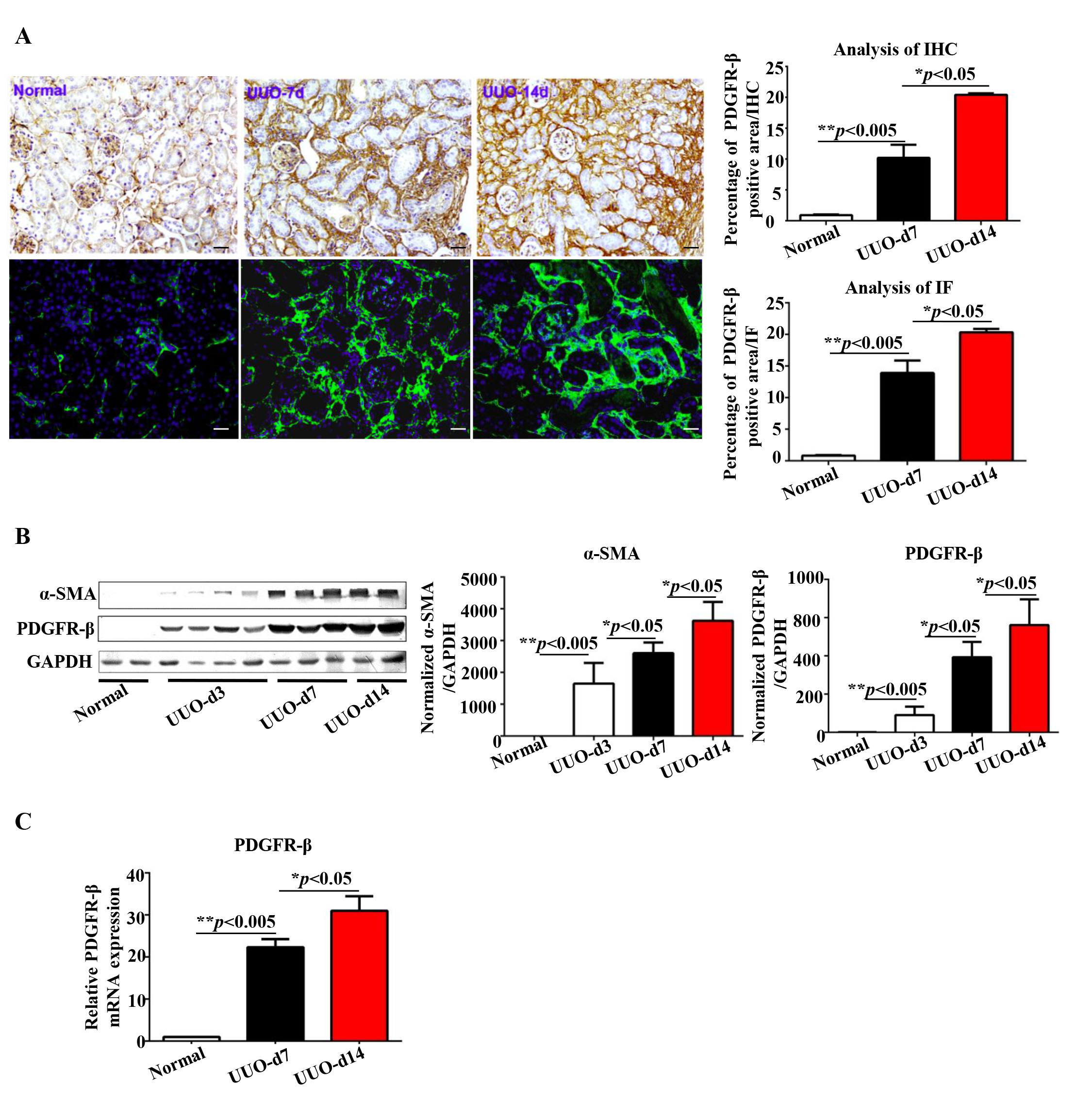

Supplement: Supplementary file 2 — Figure S2. PDGFR-β and α-SMA expression increased over time in post-UUO kidneys. (A) IF and IHC staining for PDGFR-β showed an increased number of pericytes over time in the renal interstitium. (B and C) Western blotting and qRT-PCR showed significantly increased expression of PDGFR-β and α-SMA on UUO days 7 and 14. The data are presented as the mean ± SD; n = 5/group. *p < 0.05; **p < 0.005. (TIF 19464 kb) [file 13287_2019_1201_MOESM2_ESM.tif]

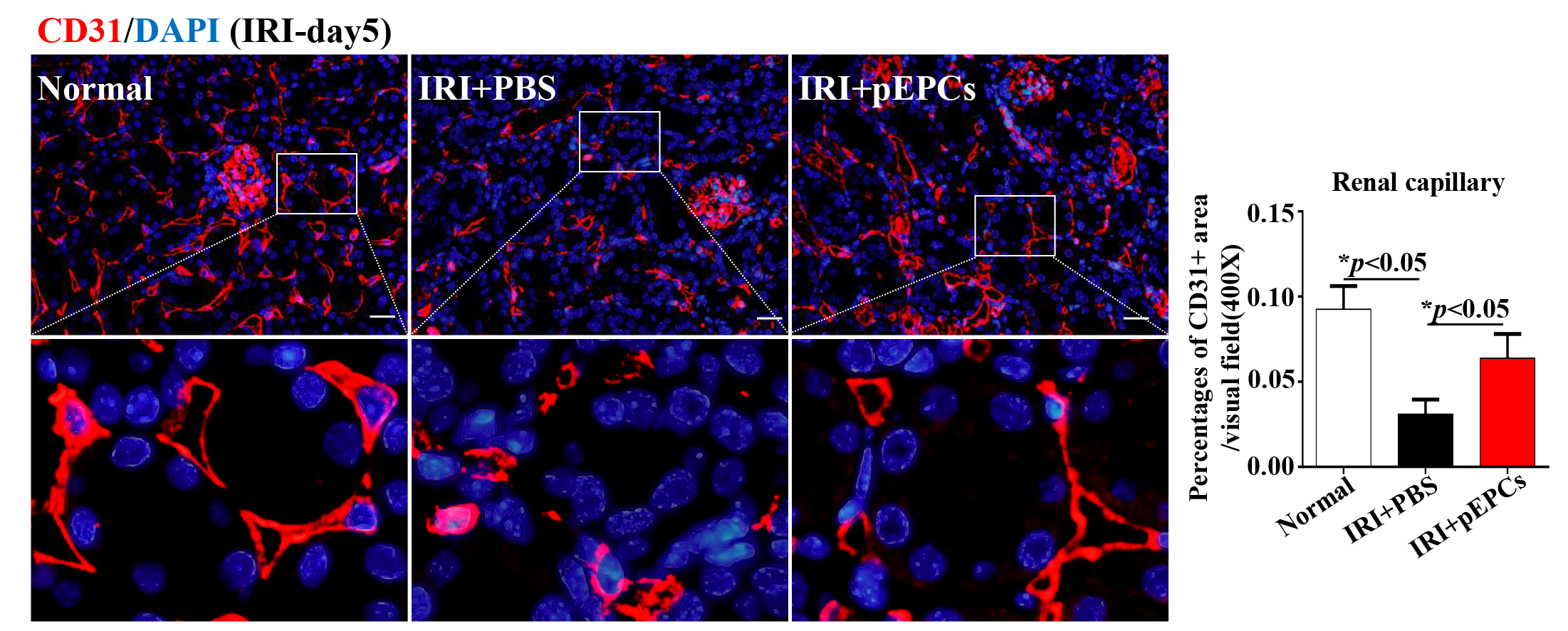

Supplement: Supplementary file 3 — Figure S3. pEPCs alleviated kidney microvascular rarefaction after ischaemia reperfusion injury. Mice were subjected to ischaemia reperfusion injury (IRI) and sacrificed on day 5. CD31 staining (red) showed that endothelial cells decreased on IRI-day 5. Exogenous injection of pEPCs promoted vascular repair and reduced capillary rarefaction. The data are presented as the mean ± SD; n = 4–5/group. *p < 0.05. Scale bar, 20 μm. (TIF 8318 kb) [file 13287_2019_1201_MOESM3_ESM.tif]
